# The impact of antibiotic exposure on antibiotic resistance gene dynamics in the gut microbiota of inflammatory bowel disease patients

**DOI:** 10.3389/fmicb.2024.1382332

**Published:** 2024-04-17

**Authors:** Yufei Zhang, Gaogao Xue, Fan Wang, Jing Zhang, Lida Xu, Changyuan Yu

**Affiliations:** ^1^College of Life Science and Technology, Beijing University of Chemical Technology, Beijing, China; ^2^Beijing Hotgen Biotech Co., Ltd., Beijing, China; ^3^Beijing YuGen Pharmaceutical Co., Ltd., Beijing, China

**Keywords:** metagenomics, gut microbiome, drug resistance, antibiotics resistance genes, horizontal gene transfer

## Abstract

**Background:**

While antibiotics are commonly used to treat inflammatory bowel disease (IBD), their widespread application can disturb the gut microbiota and foster the emergence and spread of antibiotic resistance. However, the dynamic changes to the human gut microbiota and direction of resistance gene transmission under antibiotic effects have not been clearly elucidated.

**Methods:**

Based on the Human Microbiome Project, a total of 90 fecal samples were collected from 30 IBD patients before, during and after antibiotic treatment. Through the analysis workflow of metagenomics, we described the dynamic process of changes in bacterial communities and resistance genes pre-treatment, during and post-treatment. We explored potential consistent relationships between gut microbiota and resistance genes, and established gene transmission networks among species before and after antibiotic use.

**Results:**

Exposure to antibiotics can induce alterations in the composition of the gut microbiota in IBD patients, particularly a reduction in probiotics, which gradually recovers to a new steady state after cessation of antibiotics. Network analyses revealed intra-phylum transfers of resistance genes, predominantly between taxonomically close organisms. Specific resistance genes showed increased prevalence and inter-species mobility after antibiotic cessation.

**Conclusion:**

This study demonstrates that antibiotics shape the gut resistome through selective enrichment and promotion of horizontal gene transfer. The findings provide insights into ecological processes governing resistance gene dynamics and dissemination upon antibiotic perturbation of the microbiota. Optimizing antibiotic usage may help limit unintended consequences like increased resistance in gut bacteria during IBD management.

## Introduction

Antibiotics, capable of killing or inhibiting pathogenic bacteria, have been widely used for treating and preventing bacterial infections in humans and other animals (Cook and Wright, 2022), representing one of the indispensable clinical tools (Kanj et al., 2022). However, their widespread usage has led to diminishing efficacy against an increasing array of bacterial pathogens (Patangia et al., 2022), leading to increased morbidity and mortality (Tillotson and Zinner, 2017; Lerminiaux and Cameron, 2019). Alarmingly, current estimations indicate antibiotic resistance kills over 700,000 people annually worldwide (Lerminiaux and Cameron, 2019). Moreover, within the next three decades, drug-resistant infections could claim 10 million lives per year, exceeding the number of deaths from cancer (Mudenda et al., 2023). For patients with inflammatory bowel disease (IBD), whose long-term antibiotic exposure for IBD management (Khan et al., 2011; Nitzan et al., 2016), can increase the risk of developing drug resistance, this may potentially constrain treatment choices and exacerbate difficulties in care (Ledder, 2019). Therefore, it is necessary to investigate the underlying mechanisms by which antibiotic misuse leads to bacterial acquisition of drug resistance, especially among high risk populations like IBD patients, in order to find ways to control antibiotic resistance.

The human gut microbiota forms a complex functional metabolic network through dynamic microbial interactions, which plays an important role in maintaining health (Cheng et al., 2019; Hertli and Zimmermann, 2022). The gut microbiota is not only a sophisticated ecosystem, but also an important reservoir of antibiotics resistance genes (ARGs) (Hu et al., 2013; Hofer, 2022). Several metagenomic analyses have revealed that the structure of the gut microbiota is markedly influenced by these resistance genes (Dhariwal et al., 2023), with a shared network of ARGs existing between pathogenic and commensal gut microorganisms (Forster et al., 2022). In order to solve the increasingly severe problem of drug resistance, we must understand how bacteria acquire and disseminate resistance genes in clinical settings. Notably, higher abundances of ARGs have been detected in the gut microbiota of IBD patients compared to healthy controls (Fredriksen et al., 2023). Understanding the mechanisms of antibiotic resistance gene dissemination within gut microbiota is critically important for controlling and preventing the development of antibiotic resistance.

Antibiotics exert a strong selective pressure on microbial populations, altering the composition and diversity of the gut microbiota (Grech et al., 2021; McDonnell et al. 2021; Patangia et al., 2022). Antibiotic treatment can select for resistant pathogens that cause subsequent infections (Ianiro et al., 2020; De Nies et al., 2023), and antibiotics targeting specific species may also act on commensal species in patients (Korpela et al., 2016; Yang et al., 2021), thereby collectively disrupting microbial community stability. Disturbances to the stability of the gut microbiota could reduce colonization resistance against invading pathogens (Wu and Wu, 2012; Becattini et al., 2016), and increase the risk of other diseases (Zheng et al., 2020). Particularly in IBD patients, the gut microbiota is already in an unstable state due to the disease condition (Clooney et al., 2021; Kharaghani et al., 2023). Prolonged antibiotic use may further intensify imbalances in the community by increasing selective pressures for resistant strains (Rashid et al., 2015; Cižman and Plankar, 2018), potentially exacerbating antibiotic resistance in IBD patients (Park et al., 2014; Nitzan et al., 2016).

Such antibiotic-induced changes in the gut microbiota have been associated with an increase in ARGs (Pan et al., 2022). Dhariwal et al. (2023) showed that long-term antibiotic use sustains elevated levels of resistance genes, with these change enduring beyond the alterations in gut bacteria composition. Antibiotic selection may lead to accumulation of ARGs among commensal bacteria. Kathryn et al. (Winglee et al., 2017) discovered through metagenomic sequencing that the diversity of ARGs in the gut microbiota increases with the intensity of antibiotic treatment. Therefore, we need to investigate how antibiotics facilitate the spread of resistance genes within the gut microbiota.

Horizontal gene transfer (HGT) is a critical mechanism enabling bacteria to acquire new ARGs beyond their clonal evolutionary lines, thereby enhancing their antibiotic resistance (Tao et al., 2022). Jiang et al. (2017) provided evidence for the transfer of ARGs from actinobacteria to proteobacteria. Under the pressure of antibiotics, HGT fosters genomic diversity and rapid adaptation in bacteria (Ramamurthy et al., 2022). Antibiotics can amplify the dissemination of ARGs among gut bacterial communities, with profound and lasting impacts on microbial composition (Jutkina et al., 2016; Dhariwal et al., 2023). The enrichment of mobile ARGs in certain bacterial phyla (Hu et al., 2016) and the primary dissemination of resistance within the same genus or between different phyla (De Abreu et al., 2021) through genetic exchanges are notable findings. Particularly, a large number of horizontal gene transfer (HGT) phenomena were observed in IBD patients (Li et al., 2020), affecting the microbiome composition. Current research, however, lacks comprehensive understanding of ARGs transmission within the human gut microbiota before and after antibiotic use, making it crucial to characterize the resistome in this environment for developing personalized antimicrobial management strategies.

This study aims to investigate the impact of antibiotic treatment on the distribution and transfer of ARGs within the human gut microbiota. Mobile antibiotic resistance genes are particularly prevalent among patients with IBD (Li et al., 2020). Individuals with IBD are often administered antibiotic therapy (Nitzan et al., 2016) which can induce the horizontal transfer of ARGs within the intestinal microbiota. Based on this, we Utilizing metagenomic data from IBD patients in the Human Microbiome Project (HMP2), examining fecal samples at different stages of antibiotic therapy. Through analyzing the metagenomic sequences and interaction networks, we characterized the resistome and the pathways of ARG transfer induced by antibiotics. Our research seeks to elucidate the ecological processes governing ARG dynamics in the gut, potentially aiding in optimizing antibiotic usage and minimize unintended drug resistance promotion in IBD management.

## Methods

### Data sources

Metagenomics are capable of identifying new taxonomic groups (Wang et al., 2020) and resistance genes, which offers insights into microbial communities and resistance mechanisms (Li et al., 2021), making it a valuable tool in this research. This study used the metagenomic data from the Infectious Bowel Disease Multi-‘omics Database (IBDMDB) project of HMP2 (Lloyd-Price et al., 2019), focusing on patients treated with antibiotics (Dubinsky et al., 2020). The IBDMDB project contains 1,638 metagenomic samples from 130 patients, of which 54 received antibiotic treatment. Since the use of antibiotics for more than 10 days can significantly affect the intestinal flora (Jakobsson et al., 2010; Rashid et al., 2015), patients taking antibiotics for less than 2 weeks were excluded, which left 30 patients. Samples collected more than 30 days after antibiotic cessation were considered “pre-treatment” as most microbial communities require around a month to fully recover from antibiotics (Dethlefsen et al., 2008). Patients taking antibiotics for 14 days represented the “during-treatment” period. Those stopping antibiotics for at least 2 weeks post-cessation were categorized as “post-treatment.” After thorough quality control, 90 total samples from the 30 patients remained. These raw data were downloaded from the Sequence Read Archive (SRA) BioProject PRJNA398089, using aspera software and subsequently transferred to a linux server for analysis.

### Sequence processing

The metagenomic sequence processing involved several stages: initial quality control, filtering, host contamination removal and a final quality control. Kneaddata pipline was used for these tasks, which integrates tools such as FastQC for quality assessment, Trimmomatic (Bolger et al., 2014) for trimming and filtering, and Bowtie2 (Langmead and Salzberg, 2012) for aligning sequences to the host genome and removing host-specific sequences. Post-processing, another round of quality control using FastQC was performed to ensure the integrity of metagenomic sequence preprocessing.

### Microbial analysis

Taxonomic assignment of metagenomic DNA sequences was conducted using Kraken software, which employs exact k-mer matching against the NCBI database and a lowest common ancestor (LCA) algorithm for rapid and accurate classification. Outputs included abundance counts for each taxon. Subsequent data processing and analysis were performed using R and Python. Specifically, the pandas package in Python was used for data manipulation, while alpha and beta diversities were calculated using the vegan package in R. Visualizations were created using matplotlib in Python and ggplot2 in R. Additionally, network analysis was conducted using the networkX package in Python, with visualization generated in Cytoscape.

### Antibiotics resistance gene analysis

Open reading frames (ORFs) were predicted from preprocessed contigs, selecting genes with a nucleotide length of ≥100 bp. These genes were clustered using CD-HIT to create a non-redundant gene set, represented by the longest sequence in each cluster. High-quality reads from each sample were mapped against this non-redundant gene set using SOAPaligner to determine gene abundances.

The Comprehensive Antibiotic Resistance Database (Alcock et al., 2023) (CARD) was utilized for antibiotic resistance gene identification. Resistance Gene Identifier (RGI) software was used to align target species gene sequences against the CARD, annotating resistance gene functionality and obtaining resistance gene annotations.

### Determination of HGT of ARGs

The BLAST method is a powerful tool used to identify species by comparing DNA or protein sequences to databases of known sequences. A 99% nucleotide identity threshold was used to determine potential horizontal gene transfer of mobile antibiotic resistance genes between species pairs based on pre- and post-treatment sequencing data (Hu et al., 2016). Specifically, if a resistance gene region displayed 99% or greater identity between the two species present before and after antibiotic use, it would suggest this gene recently transferred horizontally between these species over the treatment period.

### Statistical analysis

For the significantly different species wad used the nonparametric factorial Kruskl–Wallis test, *p* < 0.05 was considered as a significant difference. Omnibus testing was performed based on Bray Curtis distance matrices. The Bray Curtis distance matrices were derived from microbial relative abundance matrices and gene TPM matrices.

## Results

### Alterations in gut microbiota of IBD patients before and after antibiotic treatment

**Figure 1:**
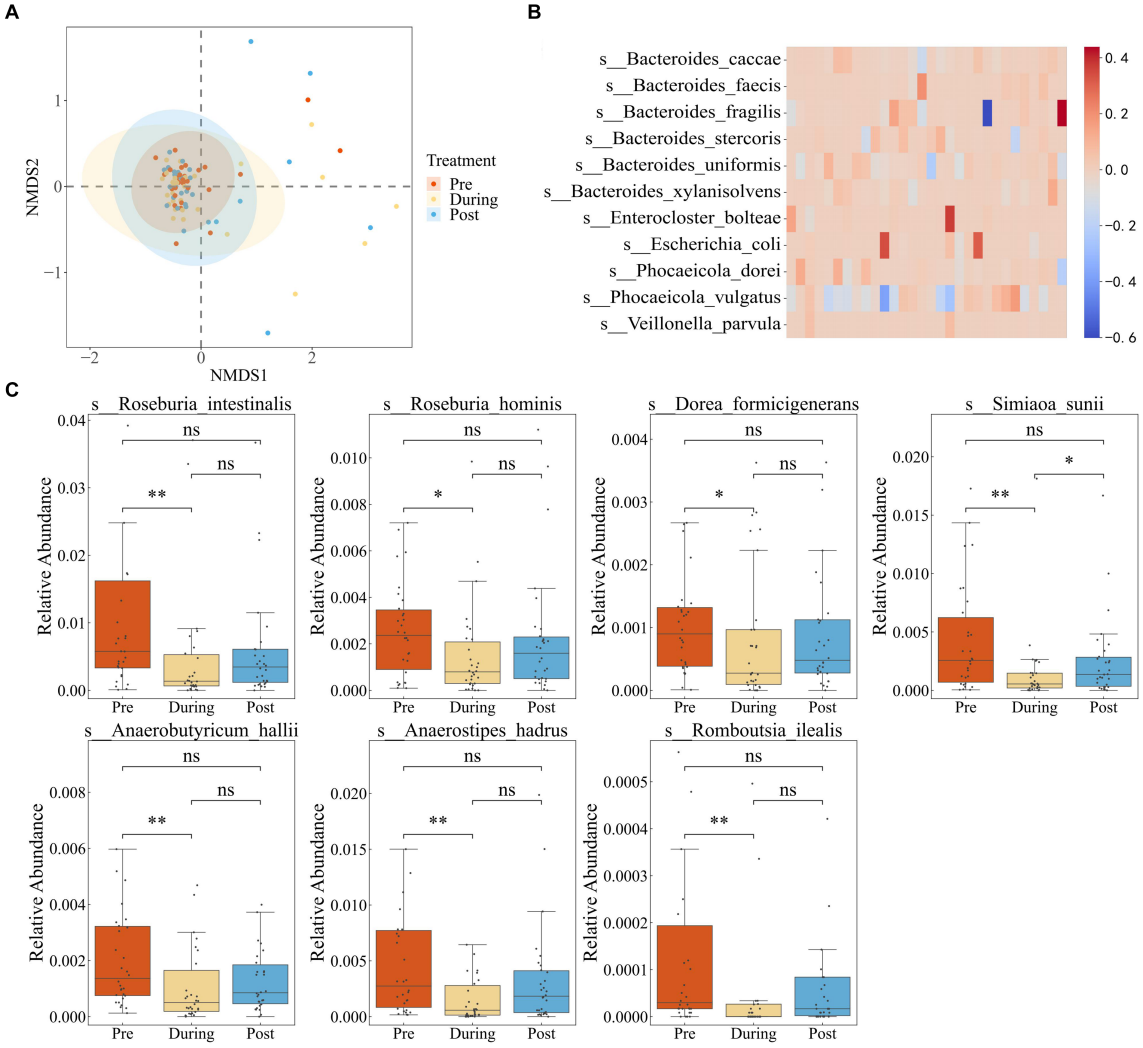
Impact of antibiotics on fecal microbiota diversity and composition in IBD patients. **(A)** NMDS based on the Bray–Curtis distance metric transformed species abundance matrix. Each point represents the bacterial microbiome of an individual sample. Colors indicate different time points. Ellipses represent 95% confidence intervals (CI) around the group clustered centroid. **(B)** Subtraction of relative abundance of dominant bacteria before and after medication. **(C)** Relative abundance of differential bacteria in three periods.

First, we performed quality control on the data to ensure accuracy in subsequent analyses (Supplementary Table S1). We employed Non-metric Multidimensional Scaling (NMDS) to examine shifts in the gut microbial community composition of IBD patients due to antibiotic treatment. In Figure 1A, the microbial communities prior to antibiotics displayed a tight clustering, indicating similar community compositions across samples. During treatment, a notable dispersion occurred, reflecting increased community divergence. But the post-treatment samples regrouped, ultimately reaching a different steady state compared to the initial clustering.

We defined dominant species as those with a relative abundance exceeding 0.1 in at least two samples (Dhariwal et al., 2023). By comparing the pre-treatment and post-treatment relative abundances of dominant species, six species showed a consistent increase in abundance after treatment in the majority of individuals. These species included *Bacteroides fragilis*, *Bacteroides stercoris*, *Bacteroides uniformis*, *Escherichia coli*, *Phocaeicola dorei*, and *Phocaeicola vulgatus* (Figure 1B). The findings suggest that the increased species potentially possess a higher abundance of resistance genes or have gained increased resistance capabilities.

To further investigate the impact of antibiotic usage on the gut microbiota of IBD patients, we performed LEFSE analysis (Supplementary Figure S1), which revealed that the majority of differentially abundant taxa belonged to the *Roseburia* genus. *Roseburia* is known for its involvement in the breakdown and fermentation of dietary fibers, producing beneficial short-chain fatty acids crucial for maintaining gut health. Relative abundance boxplots illustrated a significant decline in short-chain fatty acid-producting bacteria during the antibiotic treatment period, including *Dorea formicigenerans*, *Roseburia intestinalis*, *Anaerobutyricum hallii*, *Ruminococcus torques*, *Anaerostipes hadrus*, *Romboutsia ilealis*, and *Roseburia hominis*, with partial recovery after treatment cessation (Figure 1C). These observations highlighted that while antibiotic usage reduced the proliferation of pathogenic microbes associate with inflammatory bowel disease, it also disrupted the growth of beneficial bacteria.

Supplementary Figure S2A illustrates the predominant taxa at the phylum level in the intestinal microbiota of IBD patients, including *Bacteroidetes*, *Firmicutes*, and *Proteobacteria*. Further examination at the genus level reveals that *Bacteroides* and *Phocaeicola* were the predominant genera (Supplementary Figure S2B). Following antibiotic administration, significant variations were observed among patients, with some genera consistently responding to antibiotic treatment. Specifically, the abundance of *Bacteroides*, *Bifidobacterium*, and *Phocaeicola* increased, while *Faecalibacterium* and *Alistipes* exhibited a relative decrease. These findings suggest a degree of common response of specific bacterial genera to antibiotic exposure.

### Microbial co-occurrence network analysis

**Figure 2:**
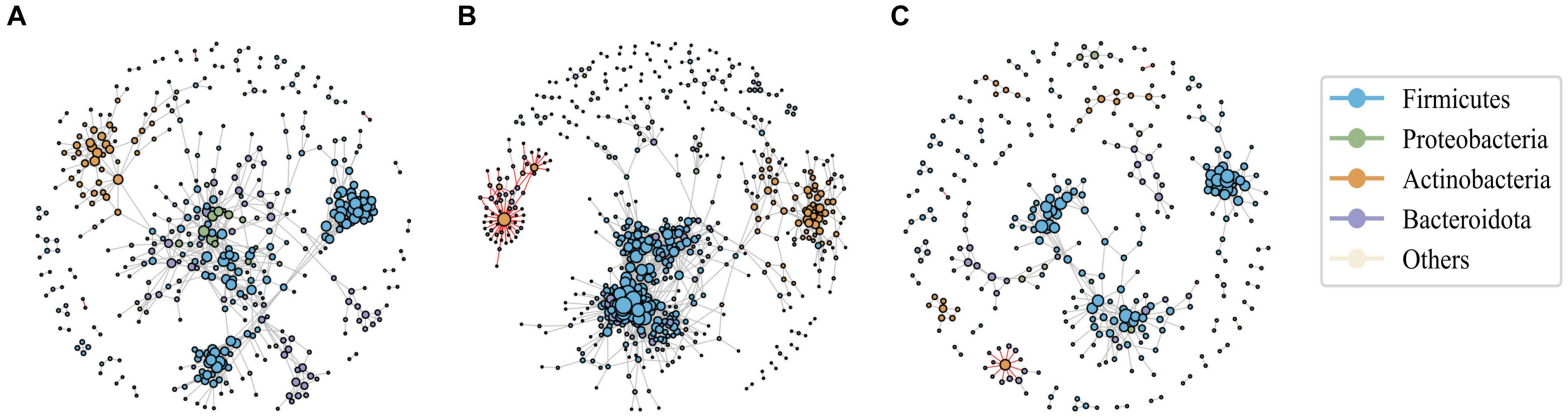
The association network of intestinal bacteria in pre-antibiotic usage **(A)**, during antibiotic usage **(B)**, and post-antibiotic cessation **(C)**. Each circle (node) represents a bacterial species, its color represents the bacterial phylum it belongs to and its size represents the number of direct edges that it has. The gray edge is positively correlated and the red edge is negatively correlated. Only significant correlations (−0.7 < *r* < 0.9, *p* < 0.05) are displayed.

Network analysis is a widely employed approach to investigate microbial interactions and to understand the changes in community structure. Here, microbial co-occurrence networks of Operational Taxonomic Units (OTUs) were constructed based on the Spearman correlation coefficient exceeding 0.9 for pre-antibiotic, during, and post-antibiotic phases (Figures 2A–C). By applying the edge-weighted spring embedded layout to the network graph, nodes with high connectivity were placed together through clustering.

The results revealed that positive correlations predominantly governed the interactions among microbial species within the networks. Compared to the baseline, the number of nodes, number of edges of the co-occurrence network in the post-antibiotic stage were significantly reduced (Supplementary Table S2), leading to a noticeable reduction in the co-occurrence network complexity in the post-antibiotic phase, indicating a decrease in the intensity of interactions. Remarkably, during treatment network complexity increased, as evidenced by the increased number of nodes, number of edges and clustering coefficient, suggesting heightened bacterial correlations, likely due to the horizontal transfer of drug resistance genes among diverse microbial populations.

### Intestinal resistance gene dynamics after antibiotic treatment

**Figure 3:**
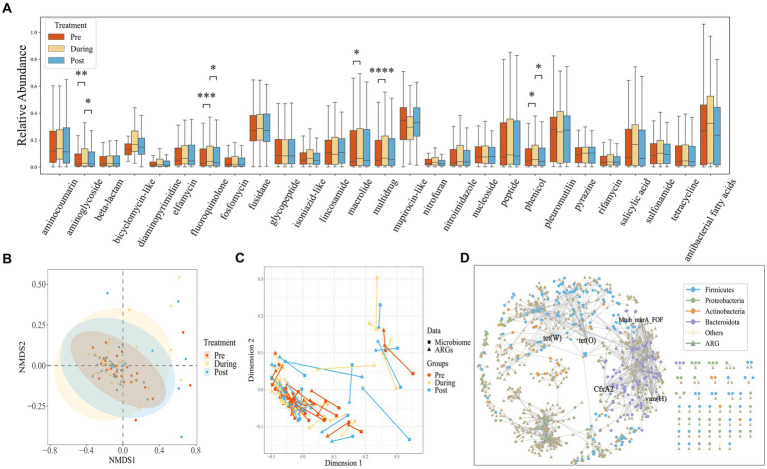
**(A)** Box plots showing the relative abundance measured as Transcript per Kilobase per Million mapped reads (TPM) of drug resistance gene (ARG) classes across all samples, stratified by time points. The center horizontal line of box is median, box limits are upper and lower quartiles, whiskers are 1.5× interquartile ranges. **(B)** NMDS based on the Bray–Curtis distance metric transformed gene TPM matrix. Each point represents the bacterial microbiome of an individual sample. Colors indicate different time points. Ellipses represent 95% confidence intervals (CI) around the group clustered centroid. **(C)** Procrustes analysis of resistome composition (filled triangles) and species composition (filled circles) of ibd patients at three time points using PCoA ordination. The points are colored based on sampling time points in both groups. The length of line connecting two points indicates the degree of dissimilarity or distance between microbiome and resistome composition of the same sample. **(D)** The network of ARGs shared among species.

Using CARD annotations, we identified a total of 1,147 distinct ARGs, encompassing 28 different antibiotics and 12 resistance mechanisms. As depicted in Figure 3A, the mupirocin-like antibiotic class exhibited the highest abundance of associated resistance genes, followed by fusidane antibiotics, pleuromutilin antibiotics, and antibacterial free fatty acids. We also found that after antibiotic treatment, the abundance of antibiotic resistance genes corresponding to aminoglycoside, fluoroquinolone, macrolide, multidrug and phenicol classes significantly increased. Regarding drug resistance gene diversity, similar to the microbial composition, the NMDS also demonstrated a shift from clustering to dispersion before eventually reaching a new stable state (Figure 3B).

Procrustes Analysis, commonly employed to assess the congruence between environmental factors and community relationships, was applied in this study. As demonstrated in Figure 3C, a significant congruence relationship was observed (Protest: sum of squares (M^2^) = 0.278, *p* = 0.001; permutations = 999), indicating that drug resistance genes can influence changes in community structure. The lengths of the lines connecting the two points represent the degree of dissimilarity between the microbial and drug resistance gene compositions of the same samples. As shown in the Figure 3C, the length of the lines between the microbiome and the drug resistance genome in the pre-treatment phase is shorter than that of the post-treatment phase, indicating that the dissimilarity between the microbial composition and drug resistance genes was lower in the pre-treatment phase than that of the post-treatment phase. This suggests that the impact of antibiotic usage on drug resistance genes may operate independently of changes in microbial composition.

To investigate the distribution of genes within species, we constructed a shared gene network among species (Figure 3D). Nodes in the network represent genes and species, while edges indicate the presence of a gene within a species. Genes found in over 20 species are labeled, and the network encompasses a total of 374 species and 703 drug resistance genes. Notably, the phyla Bacteroidetes and Proteobacteria contain the highest number of drug resistance genes. Specific genes such as tet(W), Mtub_murA_FOF, CfxA2, vanHA, and tet(O) are widely distributed across species, present in more than 20 different species.

### ARG transfer network analysis

**Figure 4:**
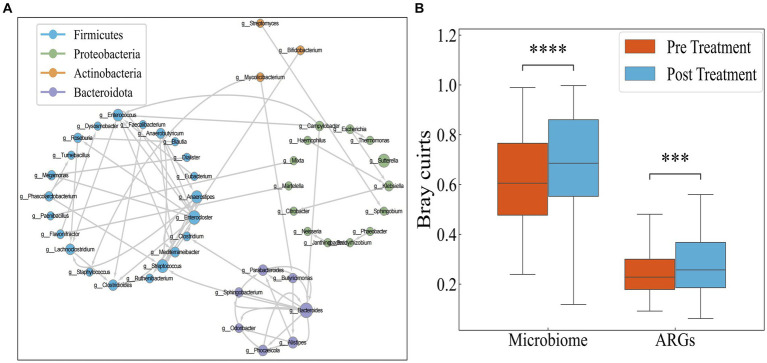
**(A)** Transmission network of ARGs in species. Each circle (node) represents a bacterial species, its color represents the bacterial phylum it belongs to and its size represents the number of direct edges that it has. **(B)** Distribution of Bray–Curtis dissimilarity of individual bacteria.

Since conjugation is the most important means of HGT of antibiotic resistance genes, and antibiotics can promote the transfer of resistance genes, we performed a BLAST analysis on sequences containing ARGs obtained before and after antibiotic treatment. If a gene exhibited differing species assignments between the pre-treatment and post-treatment BLAST results, it indicated gene transfer between these two species. Using this method, we constructed a dissemination network (Figure 4A) depicting the spread of drug resistance genes among species across 30 IBD patients.

The network encompasses a total of 46 species and 74 edges. The quantity of transferred genes is presented in Table 1, revealing that the transfer of drug resistance genes predominantly occurs within the same phylum. Notably, the phyla *Firmicutes*, *Bacteroidetes*, and *Proteobacteria* are prominently involved in the dissemination of drug resistance genes.

**Table 1:** ARG transfer across various phylums in Transmission network of ARGs depicted in Figure 4A.

Phylum before HGT	Phylum after HGT	Gene counts	Gene name
p__Bacteroidota	p__Bacteroidota	15	tet(Q), MCR-3.38, Mtub_murA_FOF, Ecol_murA_FOF, adeA, mel, Mef(En2), vanH, vanN, CfxA4, APH(4)-Ia, ErmF, ANT(9)-Ia, SPR-1, lnuA
	p__Firmicutes	1	mel
p__Firmicutes	p__Firmicutes	16	tet(W), tet(O), Cdif_gyrB_FLO, Saur_murA_FOF, tet(40), mefH, mtrC, vanS, PC1_blaZ, bcrA, ErmB, ECM-1, PLN-1, bcrB, dfrF, lnuC
	p__Proteobacteria	2	tetA(46), ErmG
p__Actinobacteria	p__Actinobacteria	3	Bado_rpoB_RIF, Saur_LmrS, Erm(48)
	p__Bacteroidota	1	APH(3′)-IIIa
	p__Firmicutes	3	tetB(46), APH(3′)-IIIa. SAT-4
	p__Proteobacteria	1	Ecol_parE_FLO

It is hypothesized that the antibiotics could potentially narrow the inter-individual differences among IBD patients by targeting pivotal hub species within the microbial network. Consequently, investigating the responses of IBD patients to antibiotics using Bray–Curtis distance aims to discern whether these responses are universally consistent or specific to individual cases. However, as illustrated in Figure 4B, both the microbial and drug resistance gene distances between individuals significantly increase following antibiotic administration (*p* < 0.05, Mann–Whitney test). This suggests that antibiotic usage exacerbates the inter-individual divergence in both microbial and drug resistance gene profiles.

Given the inherent individuality in both the microbial composition and drug resistance genes, we reconstructed the individualized transfer networks for drug resistance genes (Supplementary Figure S3). In this network, the line thickness denotes the quantity of shared resistance genes, and nodes represent genera. As indicated in Table 2, the vanH exhibited transfer phenomena across 13 individuals, consistently involving various species within the Bacteroides genus. This gene is associated with glycopeptide antibiotics. Subsequently, the Hpin_gyrA_FLO, conferring resistance to fluoroquinolones, demonstrated transfer in 10 individuals. This resistance gene corresponds to fluoroquinolone antibiotics, a class of broad-spectrum antibiotics.

**Table 2:** ARGs statistics of transfer in Supplementary Figure S3.

phylum_before	phylum_after	gene	Drug Class	Resistance Mechanism	Patient counts
p__Bacteroidota	p__Bacteroidota	vanH	glycopeptide antibiotic	antibiotic target alteration	13
p__Bacteroidota	p__Bacteroidota	Hpin_gyrA_FLO	fluoroquinolone antibiotic	antibiotic target alteration	10
p__Bacteroidota	p__Bacteroidota	cmeA	multidrug	antibiotic efflux	8
p__Bacteroidota	p__Bacteroidota	Mtub_murA_FOF	fosfomycin	antibiotic target alteration	8
p__Firmicutes	p__Firmicutes	tet(O)	tetracycline antibiotic	antibiotic target protection	6
p__Bacteroidota	p__Bacteroidota	ErmG	multidrug	antibiotic target alteration	6
p__Bacteroidota	p__Bacteroidota	vanN	glycopeptide antibiotic	antibiotic target alteration	6
p__Bacteroidota p__Firmicutes	p__Bacteroidota p__Bacteroidota	mel	multidrug	antibiotic target protection	5
p__Bacteroidota	p__Bacteroidota	catIII	phenicol antibiotic	antibiotic inactivation	5
p__Proteobacteria p__Bacteroidot p__Bacteroidota	p__Bacteroidota p__Bacteroidota p__Proteobacteria	APH(4)-Ia	aminoglycoside antibiotic	antibiotic inactivation	5

By constructing both global and individual drug resistance gene transfer networks, we observed that gene transfers predominantly occur within the same phylum, with the phyla *Bacteroidetes* and *Firmicutes* exhibiting the highest number of transferred genes. Moreover, the transferred genes are primarily associated with resistance to broad-spectrum antibiotics.

## Discussion

Antibiotics, while a mainstay in managing inflammatory bowel disease (IBD), also pose a risk of generating antibiotic-resistant bacteria and propagating antibiotic resistance genes (Ledder, 2019; Lerminiaux and Cameron, 2019), potentially leading to the treatment intolerance. To evaluate the impacts of antibiotics on the gut microbiota and resistome, this study analyzed the dynamic of the intestinal flora and the transfer of resistance genes by encompassing the metagenomic data through various treatment phases.

While antibiotics target pathogenic bacteria, they may also disrupt the normal gut microbiota’s balance, either directly or indirectly (Rashid et al., 2015). Typically, the majority of the microbiota revert to their pre-exposure state within 2–4 weeks following antibiotic treatment (Rashid et al., 2012, 2015). Our research align with this notion, illustrating a significant reduction in short-chain fatty acid-producing bacteria during antibiotic administration, followed by a gradual recovery after antibiotic withdrawal. This pattern indicates an inherent resilience within the microbial community (Dethlefsen et al., 2008; Jian et al., 2021). Our study found that both the intestinal microbiota and drug-resistant profile exhibited self-recovery capabilities post-antibiotic treatment, as shown in the network analyses and Procrustes analysis. Network analysis showed increased co-occurrence of taxa during antibiotic exposure, implying inter-species interactions at the genetic level such as resistance gene transfer. Procrustes analysis corroborated the potential congruence between the resistome and microbiota over time, suggesting resistance genes may affect the evolution of microbial community structure.

Previous studies have established that bacteria can exchange ARGs through various mechanisms such as conjugation, transformation and transduction, thus spreading antibiotic resistance (Munita and Arias, 2016; Tao et al., 2022). Our study, focusing on the distribution and transmission of ARGs within human gut microbiota induced by antibiotic exposure, identified Bacteroides and Proteus as having the most ARGs. While we found the GC content after treatment to be higher than before treatment, the difference was not significant (Supplementary Figure S4). This is consistent with findings in the literature (Jaramillo et al., 2015) demonstrating no clear association between GC content and horizontal gene transfer. Based on this, we constructed comprehensive and individual-level ARG transfer networks, and found that ARG transfers predominantly occurred between closely related taxa within the same phylum, hinting at a phylogenetic barrier to gene transfer (Hu et al., 2016). In the transfer networks, Firmicutes, Bacteroidetes, and Proteobacteria harbored the most of the transferred ARGs. Furthermore, consistent with our findings, a retrospective study (Kusan et al., 2022) have demonstrated that antibiotics can induce resistance development in pathogenic *E. coli*, which belongs to the phylum of Proteobacteria. Notably, genes like vanH, associated with glycopeptide antibiotic resistance (Marshall et al., 1998; Blaskovich et al., 2018) were commonly transferred in most individuals, potentially linked to vancomycin use in IBD treatment (Guinan et al., 2019; Lei et al., 2019).

Our findings underscore that antibiotic use in IBD not only alters the richness and balance of the gut microbiota, but also facilitates the spread of ARGs, posing potential risks to patient prognosis. Our results provide evidence for the ecological dysbiosis in the microbiota and resistome due to antibiotic intervention, and reveal the pathways of ARGs dissemination within the gut. To mitigate these unintended effects, future antibiotic treatment regimens for IBD could incorporate fecal bacteria transplantation with healthy patients (Keshteli et al., 2017; Cai et al., 2021). Providing beneficial microbes along with antibiotics may help restoration of microbial diversity and resistome balance after treatment (Cubillos-Ruiz et al., 2022), leading to optimized management of IBD while mitigating risks of exacerbating antibiotic resistance in the gut. However, this study’s limited sample size calls for caution in generalizing the findings, necessitating further validation with larger cohorts. Additionally, the observed horizontal gene transfers of resistance genes through bioinformatics require experimental confirmation. Furthermore, exploring the resistome-microbiota dynamics across various antibiotic classes and combinations remains an area for future research.

## Data availability statement

The original contributions presented in the study are included in the article/Supplementary material, further inquiries can be directed to the corresponding author.

## Ethics statement

Ethical approval was not required for the study involving humans in accordance with the local legislation and institutional requirements. Written informed consent to participate in this study was not required from the participants or the participants’ legal guardians/next of kin in accordance with the national legislation and the institutional requirements.

## Author contributions

YZ: Writing – original draft, Methodology, Visualization. GX: Software, Writing – review & editing. FW: Investigation, Writing – review & editing. JZ: Visualization, Writing – original draft. LX: Writing – review & editing, Investigation, Writing – original draft. CY: Methodology, Supervision, Writing – review & editing.
