# Supplementary material for: The impact of antibiotic exposure on antibiotic resistance gene dynamics in the gut microbiota of inflammatory bowel disease patients
Source: Front Microbiol. 2024 Apr 17;15:1382332. doi: 10.3389/fmicb.2024.1382332 (PMC11061493; doi:10.3389/fmicb.2024.1382332)
Supplement: Supplementary file 1 [file Table_1.docx]

**Supplemental material table S1. Data quality control table**

| **group** | **pre treatment** | **during treatment** | **post treatment** |
| --- | --- | --- | --- |
| Clean total reads(M) | 19.577 | 21.371 | 21.716 |
| Clean total bases(G) | 1.814 | 2.002 | 2.016 |
| Clean Q30 bases rate | 0.960 | 0.958 | 0.959 |
| Clean Q20 bases rate | 0.985 | 0.984 | 0.984 |
| Clean GC content(%) | 0.454 | 0.454 | 0.458 |
