# Supplementary material for: The impact of antibiotic exposure on antibiotic resistance gene dynamics in the gut microbiota of inflammatory bowel disease patients
Source: Front Microbiol. 2024 Apr 17;15:1382332. doi: 10.3389/fmicb.2024.1382332 (PMC11061493; doi:10.3389/fmicb.2024.1382332)
Supplement: Supplementary file 2 [file Table_2.docx]

**Supplementary Table S2. Topological properties of co-occurrence networks in Figure 2**

|  | Nodes | Edges | Clustering coefficient |
| --- | --- | --- | --- |
| Pre-Treatment | 427 | 949 | 0.437 |
| During-Treatment | 647 | 2847 | 0.464 |
| Post-Treatment | 323 | 534 | 0.465 |
