# Supplementary figures and images for: The impact of antibiotic exposure on antibiotic resistance gene dynamics in the gut microbiota of inflammatory bowel disease patients
Source: Front Microbiol. 2024 Apr 17;15:1382332. doi: 10.3389/fmicb.2024.1382332 (PMC11061493; doi:10.3389/fmicb.2024.1382332)

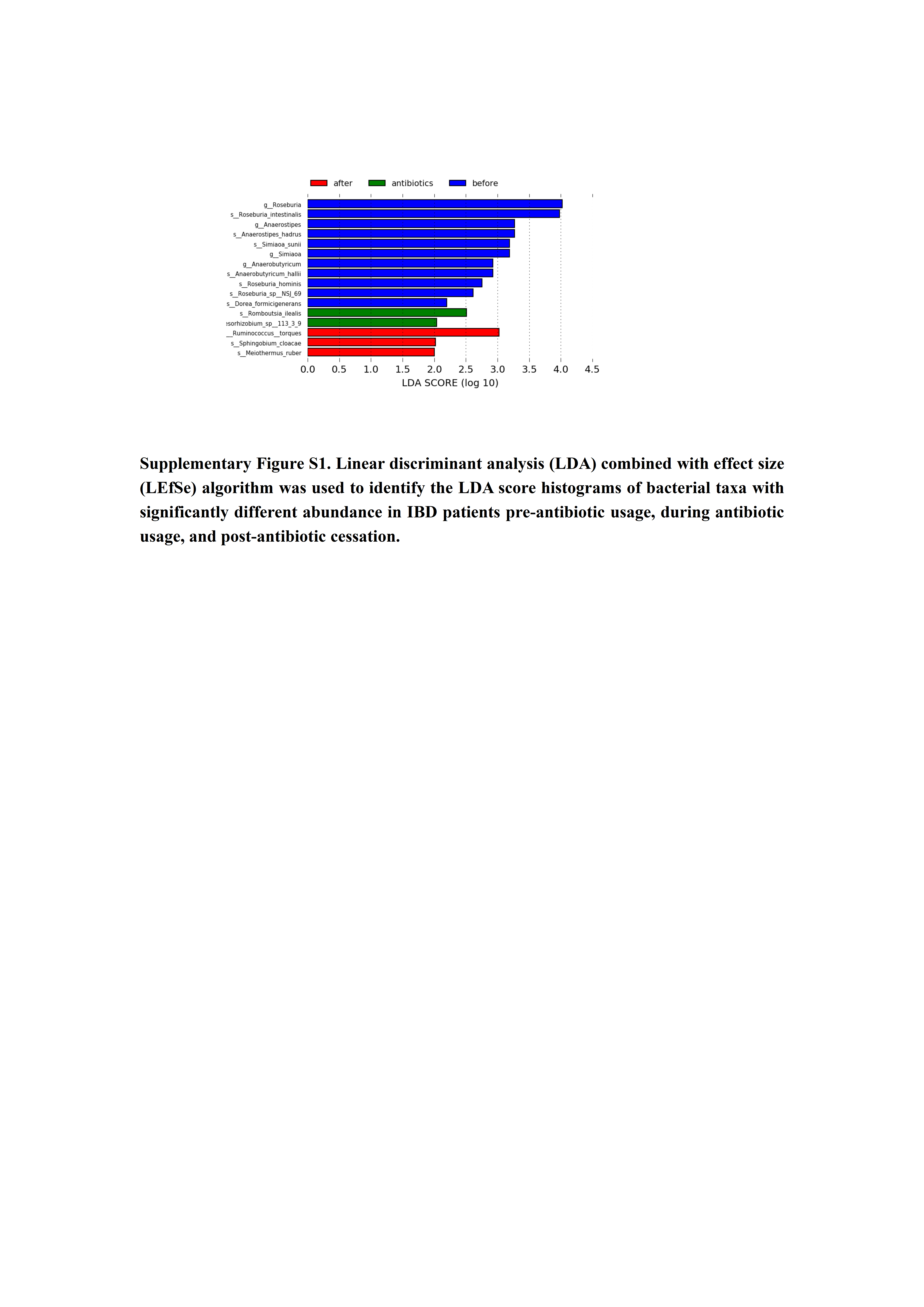

Supplement: Supplementary file 3 [file Image_1.png]

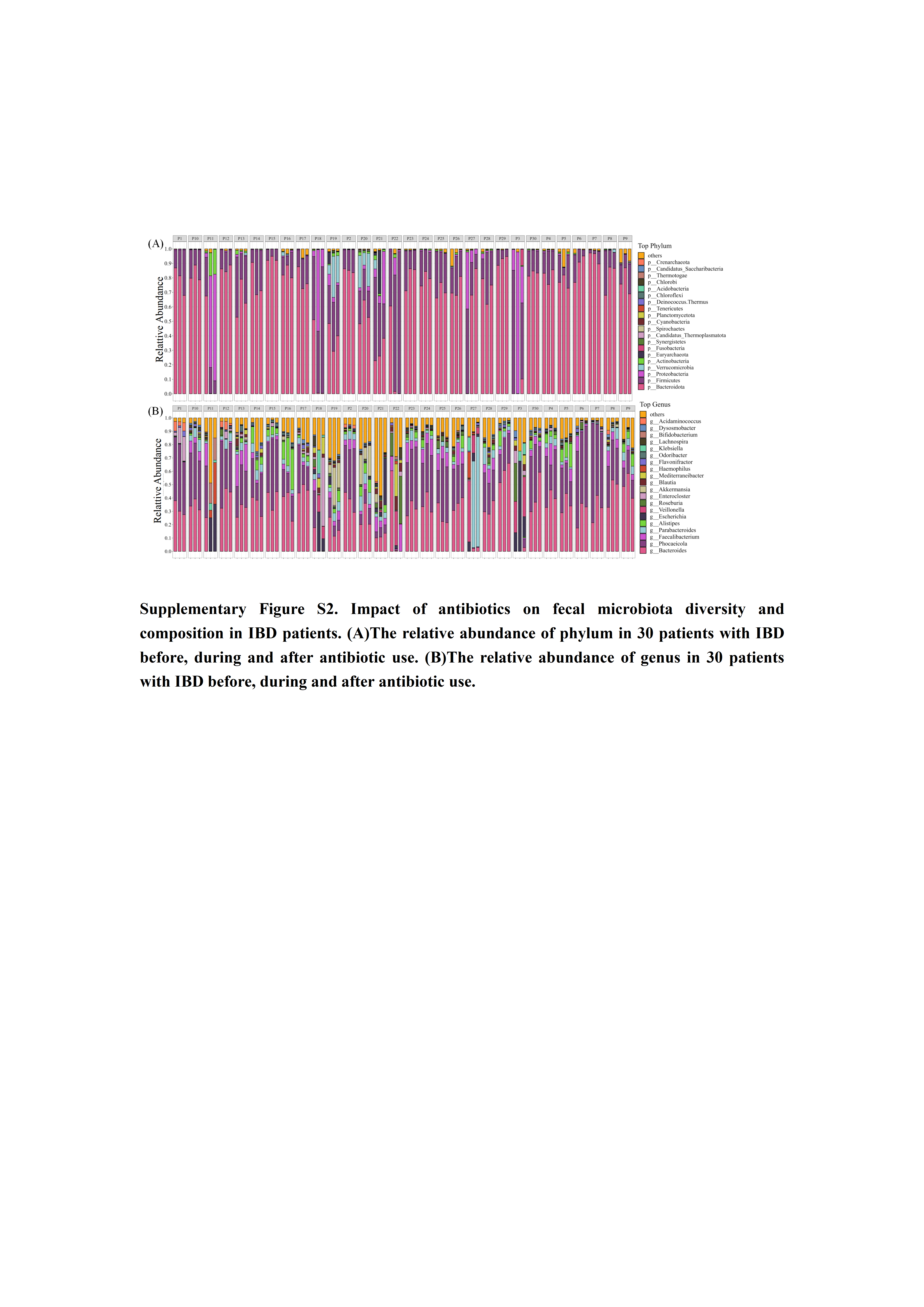

Supplement: Supplementary file 4 [file Image_2.png]

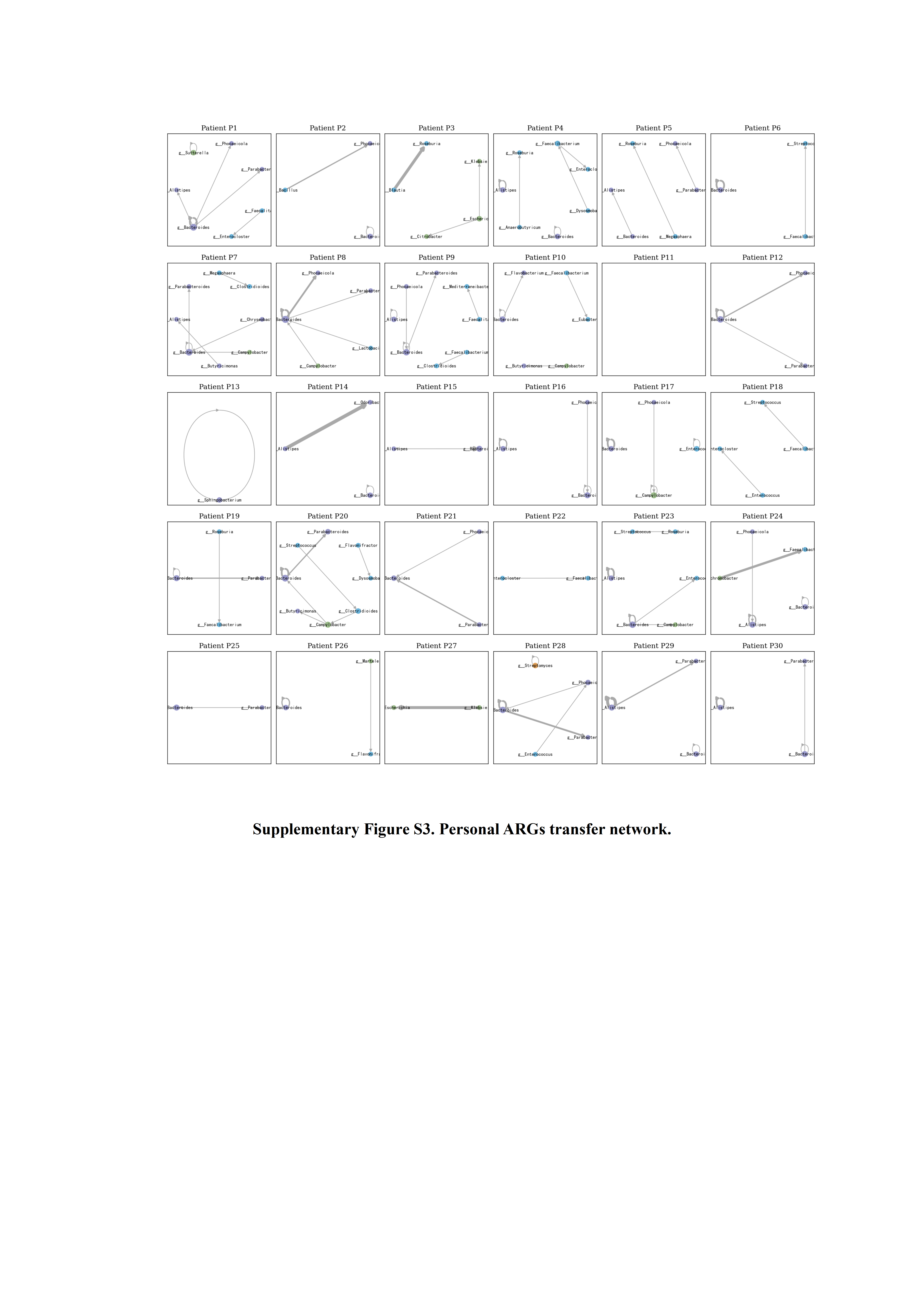

Supplement: Supplementary file 5 [file Image_3.png]

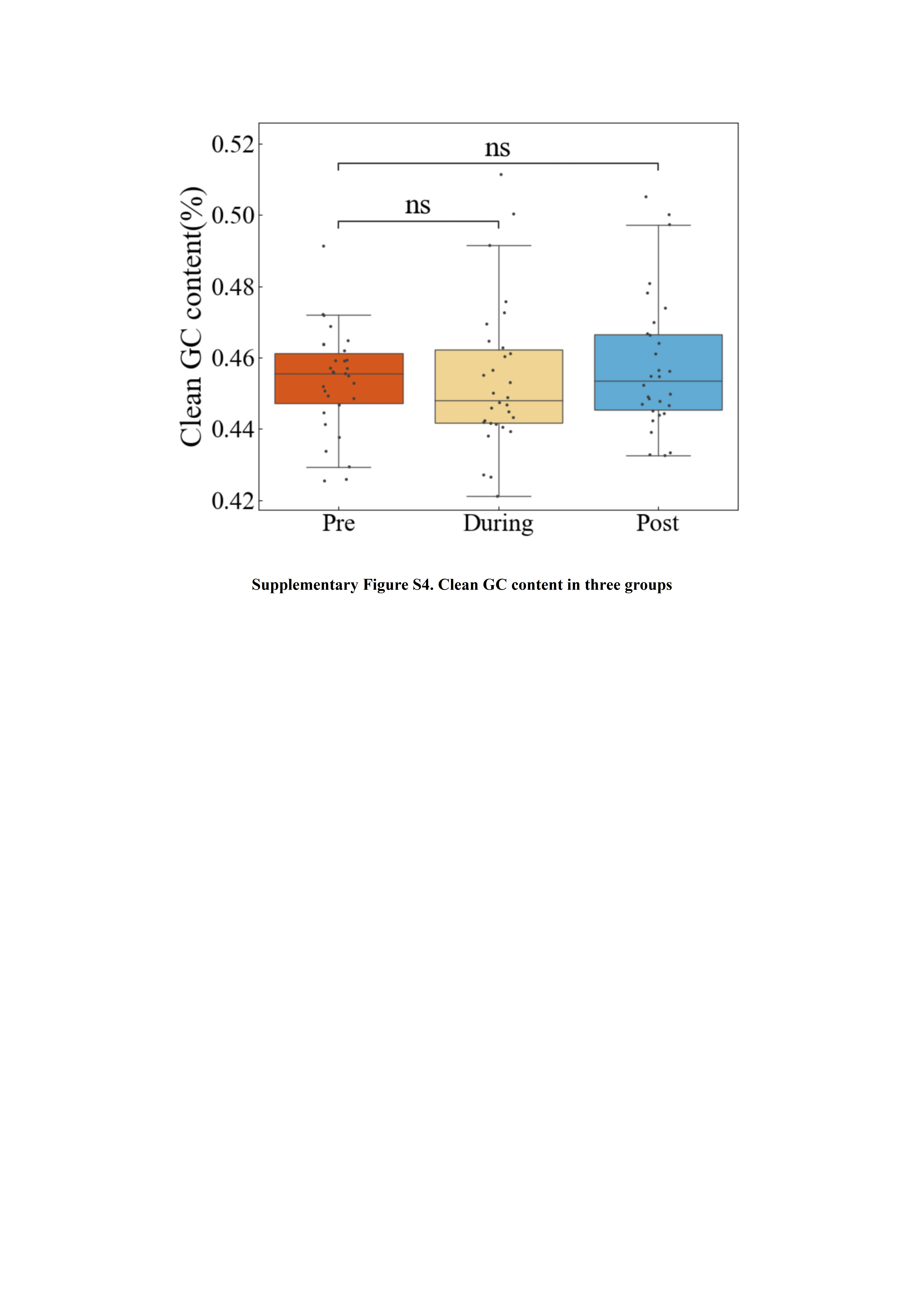

Supplement: Supplementary file 6 [file Image_4.png]
